# Intelligent prediction models based on machine learning for CO_2_ capture performance by graphene oxide-based adsorbents

**DOI:** 10.1038/s41598-022-26138-6

**Published:** 2022-12-13

**Authors:** Farnoush Fathalian, Sepehr Aarabi, Ahad Ghaemi, Alireza Hemmati

**Affiliations:** 1 Department of Chemical Engineering, Faculty of Engineering, Islamic Azad University, South Tehran Branch, Tehran, Iran; 2 School of Chemical, Petroleum and Gas Engineering, Iran University of Science and Technology, (IUST), Narmak, Tehran, 16846 Iran

**Keywords:** Environmental sciences, Engineering, Mathematics and computing

## Abstract

Designing a model to connect CO_2_ adsorption data with various adsorbents based on graphene oxide (GO) which is produced from various forms of solid biomass, can be a promising method to develop novel and efficient adsorbents for CO_2_ adsorption application. In this work, the information of several GO-based solid sorbents were extracted from 17 articles aimed to develop a machine learning based model for CO_2_ adsorption capacity prediction. The extracted data including specific surface area, pore volume, temperature, and pressure were considered as input parameter, and CO_2_ uptake capacity was defined as model response, alsoseven different models, including support vector machine, gradient boosting, random forest, artificial neural network (ANN) based on multilayer perceptron (MLP) and radial basis function (RBF), Extra trees regressor and extreme gradient boosting, were employed to estimate the CO_2_ adsorption capacity. The best performance was obtained for ANN based on MLP method (*R*^2^ > 0.99) with hyperparameters of the following: hidden layer size = [45 35 45 45], optimizer = Adam, the learning rate = 0.003, *β*_1_ = 0.9, *β*_2_ = 0.999, epochs = 1971, and batch size = 32. To investigate CO_2_ uptake dependency on mentioned effective parameters, three dimensional diagrams were reported based on MLP network, also the MLP network characteristics including weight and bias matrices were reported for further application of CO_2_ adsorption process design. The accurately predicted capability of the generated models may considerably minimize experimental efforts, such as estimating CO_2_ removal efficiency as the target based on adsorbent properties to pick more efficient adsorbents without increasing processing time. Current work employed statistical analysis and machine learning to support the logical design of porous GO for CO_2_ separation, aiding in screening adsorbents for cleaner manufacturing.

## Introduction

Up to the present, diverse physical adsorbents, like mesoporous silicates^1^, metal–organic frameworks (MOFs)^2^, carbon nanotubes (CNTs)^3^, permeable polymers^4^, and graphene^5^, have been applied to substitute conventional alkanolamines to eliminate their drawbacks, including propensity for amine misfortunes, corrosion, costly recovery, and high energy escalated^6^. Adsorption with progressed porous solid adsorbents is now beneath examination as a promising vitality and cost-efficient option^7^. The vitality required to recover solid adsorbents is ordinarily lower than that for watery amine arrangements; however, the reactivity between solid sorbent and fluid should be caught on for evaluating the ideal response enthalpy in capturing CO_2_^8^. Understanding these details could be accommodating in planning the next-generation adsorbents with lower recovery vitality requirements^9^.

Activated carbons and zeolites have been customarily utilized for gas adsorption^10^; in any case, they require considerable heating for recovery, which leads to high cost and lower efficiency^11^. Carbonaceous materials like graphene oxide (GO) are low-temperature adsorbents with specific properties, such as elevated specific surface area and reduced production price^12^. Graphene has received considerable attention nowadays^13^. The more frequent method of exfoliating graphite is to use oxidizing chemicals to produce GO, a nonporous hydrophilic carbon substance^14^. Even though the exact composition of GO is unclear, it contains epoxides, alcohols, ketone carbonyls, and carboxylic groups^15^.

GO has different applications, primarily as an adsorbent, due to its high porosity, heightened surface area, and superior chemical stability, supporting several reactive functional groups, such as hydroxyl, epoxy, and carboxyl^16,17^. Furthermore, GO is employed as an energy transformation and storage material for nanoscale engineering^18^. It is a stretchy material that delivers many possibilities for simple alteration and vision to create other preferred graphene-based substances^19^. Permeable materials can be synthesized by utilizing different methods, and their surface parameters, including surface area (*S*_*BET*_), mesopore volume (*V*_*meso*_), micropore volume (*V*_*micro*_), can be changed significantly^20^. Thus, the adsorption capacity of CO_2_ is characterized by elements such as *S*_*BET*_, porosity, isosteric heat of adsorption value (*Q*_*st*_), and the existence of micropores with a size of lower than 1 nm^21^. To enhance CO_2_ capture efficiency, extensive investigations have been committed to generate a permeable GO with increased specific surface area and pore volume. One of the most efficient methods for this purpose is functionalizing GO by amines. For example, Pokhrel et al. materialized and functionalized unique GO-based adsorbents by different amines, namely 3-aminopropyl-triethoxysilane (APTES), polyethyleneimine (PEI), and ethylenediamine (EDA)^11^. Their results can help develop optimum routes of functionalization and performance improvement of such adsorbents, paving the way for creating effective, feasible materials and methods for the forthcoming CO_2_ capture processes. Szczęsneak et al. synthesized activated carbons generated from polymers, Cu-containing metal–organic frameworks (MOFs), and their mixtures with GO for assessing their capabilities of CO_2_ adsorption under atmospheric conditions using simple procedures. Their work suggested that graphene-containing composites might be harnessed for massive CO_2_ removal under atmospheric conditions^22^. Nevertheless, optimizing and maximizing the synthesis method by mixing functionalizing agents with an acceptable guideline is still uncertain. Aside from adsorption characteristics, the textural qualities and functional groups of porous GO are commonly regarded as important CO_2_ capture factors^21^. Moreover, the method for evaluating these characteristics is unknown so far; a prioritization strategy would help support the manufacture of permeable GO-based adsorbents.

Since research facility tests are time-consuming and troublesome, a scientific forecast show is recommended. Recently, there has been a surge of attention in the use of machine learning (ML) in various domains, such as waste-to-energy conversion^23^, pyrolysis for organic and metal compound sorption^24^, methane adsorption^25^, and solid waste generated treatment^26^. Adsorption at the equilibrium state is determined by adsorbent parameters, such as surface area, pore-volume adsorbate variables (size, molecule volume, and area), the existence of functional groups, and electrostatics. It is nearly hard to get a unique correlation using a theoretical statement frame of view with the capability of properly correlating all these features concerning the equilibrium adsorption uptake^27^. Unlike the traditional isotherm models which considered only pressure and temperature as effective parameters on gas adsorption capacity, machine learning based model can consider graphene oxide textural properties such as pore size, pore diameters, surface area, pore volume, and adsorbent precursor material for GO adsorbent synthesis. Machine learning algorithms can correlate the complicated and non-linear relationships between system characteristics and adsorption uptake, this feature is the main advantage of machine learning which make it applicable in multivariable CO_2_ adsorption systems. Although machine learning based model can make relation among all of the variables which contributed to CO_2_ adsorption, but it should be considered that huge amount of data is necessary for developing the model which make some limitation for developing machine learning based model^25^.

There are different types of ML models, containing linear regression, support vector machines (SVMs), k-nearest neighbours, artificial neural networks (ANNs), and tree-based ML models. Among them, the last is a special category monitored ML methods that use iteratively numeric data division^23^. Decision trees (DTs), random forests (RF), gradient boosting decision trees (GBDTs), light gradient boosting machines (LGBs), and extreme gradient boosting (XGB) are some of the most prevalent and applicable models. Considering their novelty, the final three mentioned enhancing tree-based models have experienced a rise in popularity and applicability in scientific works due to their capacity to cope with fewer parameters, tolerance to errors, and ability to handle variable characteristics^28^. Throughout this work, the experimental specification values of several solid GO-based adsorbents, pore volumes, adsorption temperature, adsorption pressure, and BET properties were extracted and applied as inputs for training models by considering CO_2_ adsorption capacity as the target. This research aims to design algorithms that could determine the adsorption capacity of such adsorbents by applying distinctive functionalizing procedures. Furthermore, the influence of each parameter on CO_2_ uptake is examined.

High adsorption potential and selectivity, stable operating potential, expense, reusability, convenience of recovery, and fast adsorption–desorption kinetics are meticulously employed for developing adsorbents sourced from GO. Nevertheless, we mainly concentrated on the CO_2_ adsorption capacity collected at various temperatures and pressures, as well as the microstructural and morphologic properties of adsorbents, for a couple of reasons: (1) many papers have primarily focused on adsorption capacity, while only some have reported regeneration, capacity, and kinetic features; therefore, there is a limited data to present ML models for all of the stated essential properties; (2) in addition to adsorption efficiency, performance standards for other eligibility requirements were unreachable owing to the absence of ecological consequences and long-term socio analysis.

**Figure 1:**
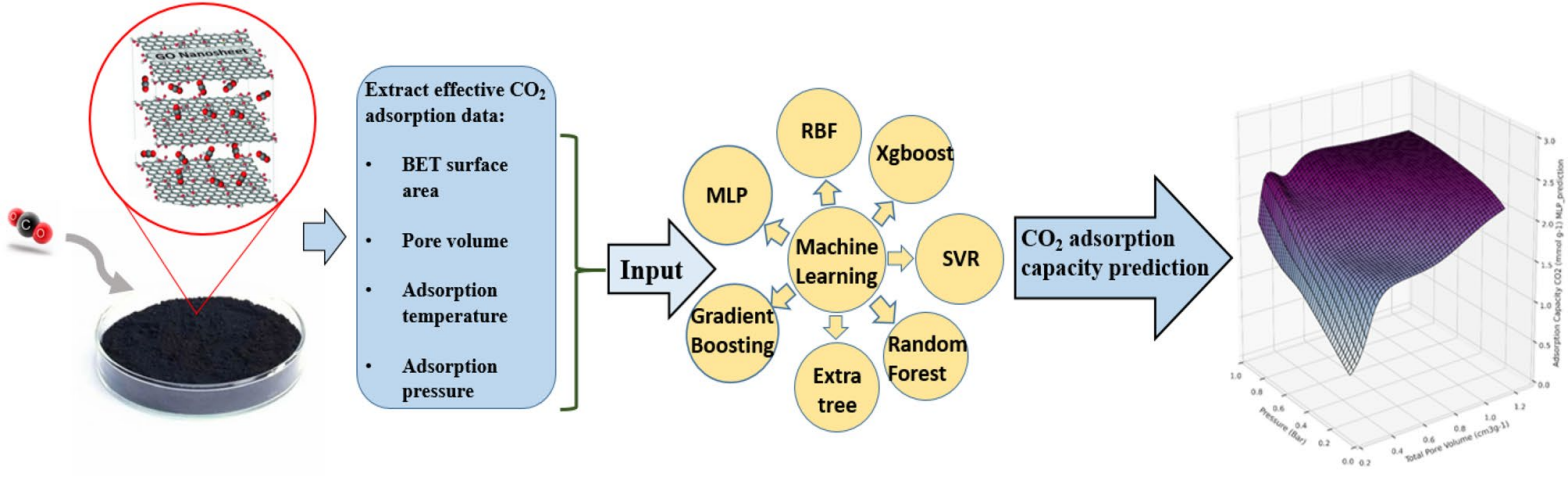
The general sketch of this research procedure.

To develop the proposed model, the following parameters and methods are implemented during the collection of information. The general sketch of this study is shown in Fig. 1.

1. All reviewed data were first approved dispassionately, with no preconceived notions or judgments about the data's trustworthiness.
2. The primary characteristics were divided into three types: (I) morphological features, (II) component compositions of the GOs, and (III) adsorption factors, including pressure and temperature (the operating pressure range was between 0 and 3 bar, and the temperature range was between 273 and 324 K) which the CO_2_ adsorption data were undertaken.
3. The GO morphological parameters consisted of specific surface area (BET, m^2^/g) and total pore volume (cm^3^/g).
4. The target parameter was CO_2_ adsorption capacity using GO-based adsorbents at various process conditions.

After a meticulous search, there is no publication available utilizing machine learning algorithms and comparing them for predicting the CO_2_ adsorption capacity of GO-based adsorbents in order to evaluate each parameter's impact on adsorption capacity.

## Data gathering and preparation

In order to gathering the CO_2_ adsorption information, a comprehensive literature study on GO-based adsorbent for CO_2_ capture was conducted using several keywords (including graphene oxide, Functionalized GO, porous carbon, CO_2_ adsorption, and CO_2_ capture). The data of textural characteristics and CO_2_ adsorption of porous carbons at 1 bar were gathered from the reported tables in each reference. The CO_2_ adsorption capacity at other pressures was obtained from adsorption isotherms using Origin Pro V9.9.0.225 Digitizer toolbox. The detailed range of operational conditions and results on GO-based adsorbents were gathered from 17 articles (895 data). Table A1 in Appendix A summarizes the sets of data obtained from the literature.

Data preparation was conducted trough examination of the papers on CO_2_ adsorption by GO-based adsorbents to create the dataset. Input characteristics were correctly detected and tagged, as supported by evidence gathered. Following information gathering, data preprocessing was undertaken to enable efficient ML deployment, and seven kinds of ML models, including SVM, Random Forest, Extra Trees, Gradient Boosting, Extreme gradient boosting, and ANN (MLP and RBF), were assessed for prediction accuracy. All of the 895 row data were acquired from the papers, and no feature was missing, so there was no requirement to correct incomplete data. It is recommended to use outlier detection models to achieve a clean data set. There are different models for outlier data detection, among them Isolation Forest has been used in this work, and the outlier data has been deleted. Isolation forest is a sort of unsupervised ML calculation that can be utilized for inconsistency detection according to the guideline of separating inconsistencies^29^. Table 1 reports the data description after removing the outliers.

**Table 1:** Detailed information of data acquired after outlier detection.

	*BET* surface area (m^2^/g)	Temperature (K)	Total pore volume (cm^3^/g)	Pressure (bar)	CO_2_ adsorption capacity (mmol/g)
Count	749.00	749.00	749.00	749.00	749.00
Mean	596.13	287.30	0.59	0.56	1.84
Std	580.83	17.70	0.41	0.30	1.83
Min	9.60	273.00	0.03	0.01	0.01
25%	99.54	273.00	0.31	0.30	0.68
50%	374.00	273.00	0.58	0.58	1.23
75%	1056.00	298.00	0.78	0.83	2.17
Max	2270.00	348.00	1.60	1.00	9.05

### Quantitative analysis of features and Pearson correlation matrix analysis

**Figure 2:**
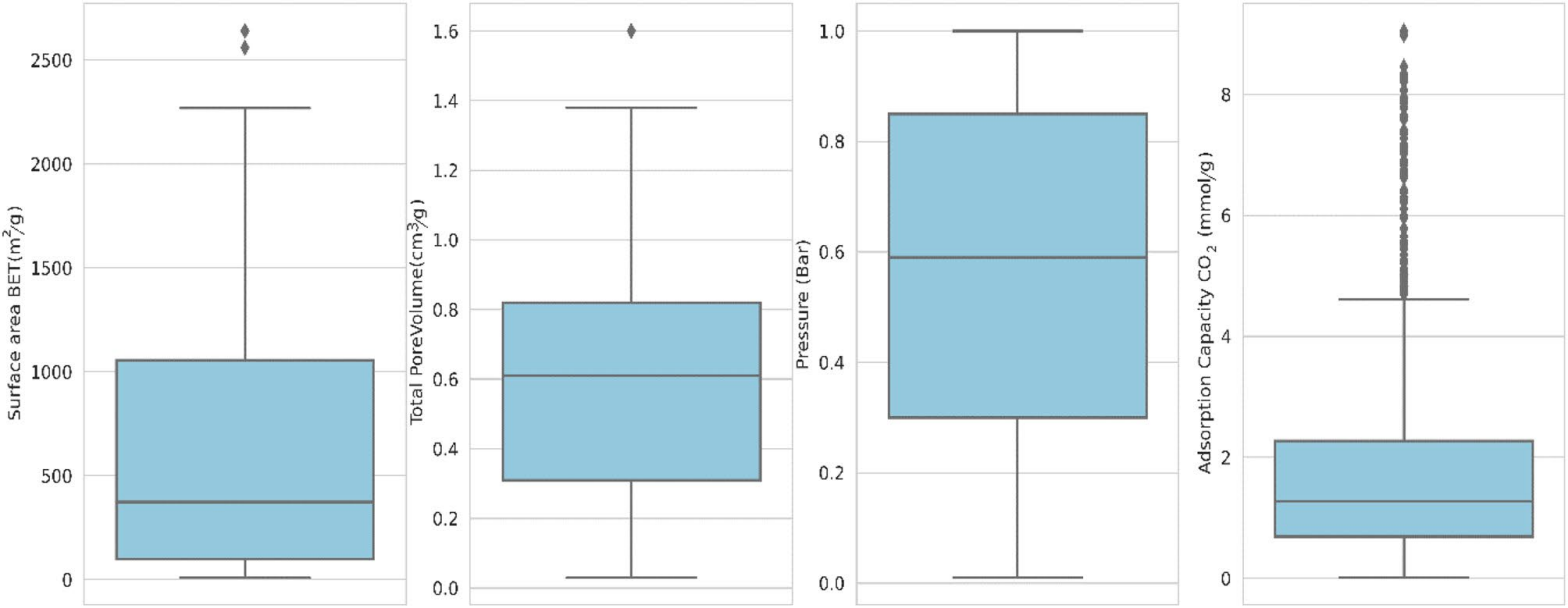
Boxplot of variables.

Statistical distribution map of each feature is shown in Fig. 2. This figure contains the structural information of the GO-based adsorbents and their related CO_2_ adsorption capacity at different pressures. The interquartile range (IQR) was used to quantify information inconsistency by partitioning the information into quartiles. Within every figure, five lines from bottom to top indicated the lowest, first quartile (*Q*_1_), middle, third quartile (*Q*_3_), and the highest statistical information, correspondingly. The stated information corresponds to the scientific results within *Q*_1_ − 1.5*IQR and *Q*_3_ + 1.5*IQR, wherein *IQR* was equivalent to the change between *Q*_3_ and *Q*_1_, and data beyond the area were displayed separately with a folded form. According to the data obtained from the study, the average value of CO_2_ adsorbed on the porous GO-based adsorbent was 1.88 mmol/g with a standard deviation of 1.82 mmol/g. The surface area determined in the study varied from 9.6 to 2640 m^2^/g, with a mean quantity of 643.47 m^2^/g and an acceptable standard deviation of 578.92 m^2^/g. Total pore volume varied from 0.03 to 1.6 cm^3^/g, with a mean value of 0.59 cm^3^/g. The treatment for modifying GO considerably affected the surface area and total pore volume, as shown in Table A1. For example, Cu-containing metal–organic frameworks (MOFs) and their GO mixtures were produced using simple techniques and evaluated for CO_2_ uptake in ambient environments. At 0 °C and 25 °C, the Cu-containing MOFs demonstrated strong CO_2_ adsorption of up to 9.59 mmol/g and 5.33 mmol/g at 1 bar, correspondingly. The analysis indicates that the surface area (*SA*) ranged from 1380 to 1820 m^2^/g and the total pore volume (TPV) ranged from 0.73 to 0.88 cm^3^/g. Furthermore, porous carbon CUBTC-GO with a *SA* of 1820 m^2^/g and a TPV of 0.83 cm^3^/g yielded the maximum CO_2_ capture of 9.05 mmol/g at 0 °C and 1 bar^22^. This conclusion showed that there was no easy and instant way to manufacture optimum porous GO for effective CO_2_ collection based on various modifications. As per the 19 publications cited here (summarized in Table A1), the investigators just chose the best CO_2_ adsorbent from several synthesized nanoparticles obtained from GO, suggesting that there was no valuable and concise advice for the formation of high CO_2_ adsorbents based on GO. Overall, the textural chracteristics of porous GOs were more important used for changing their CO_2_ adsorption capacity than chemical composititions in every considered scenario.

**Figure 3:**
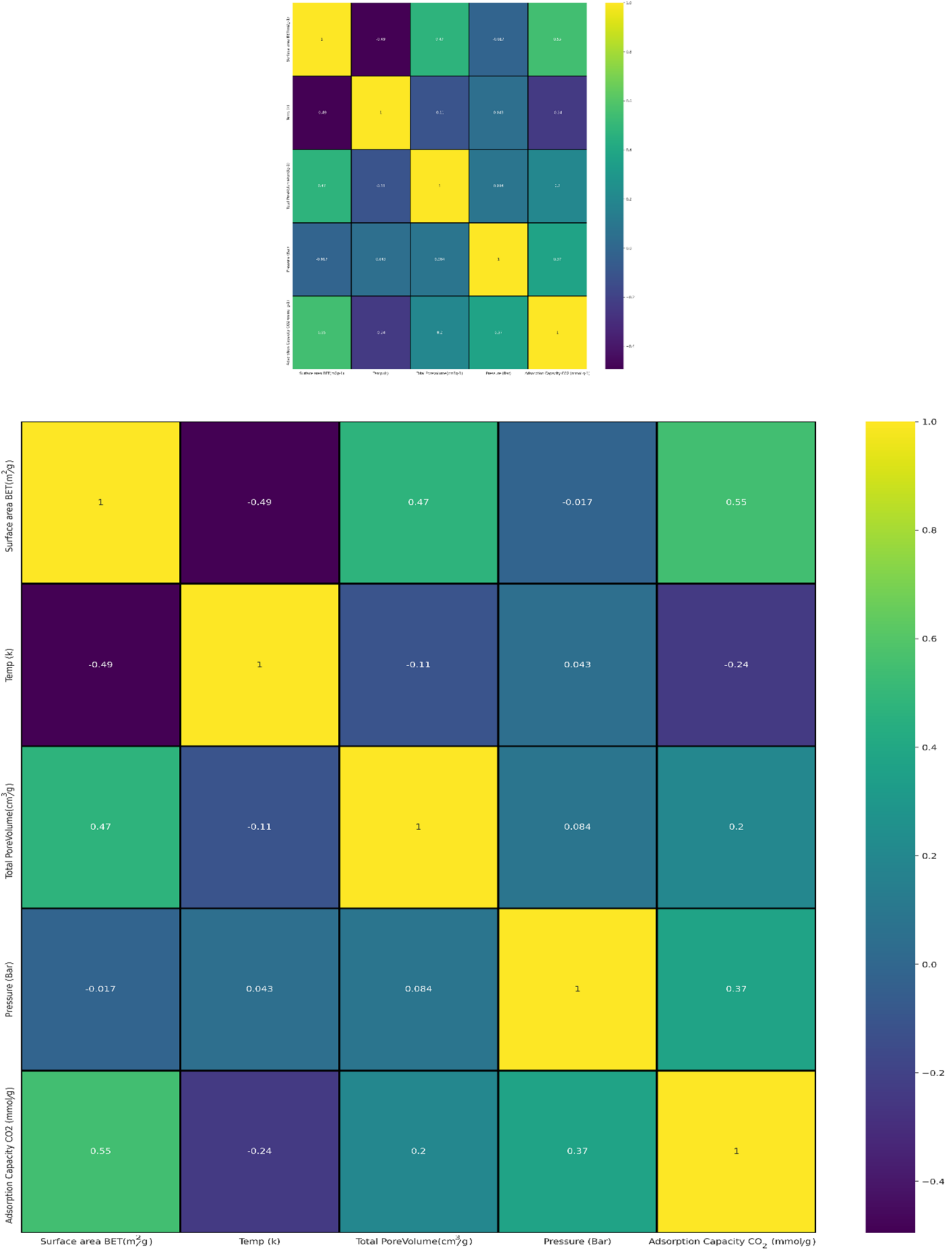
Pearson correlation matrix between any two properties, and between adsorption efficiency and each variable.

Pearson correlation coefficient matrix is the covariance of two mentioned feature and the product of their standard deviation. According to pearson correlation coefficient matrix which represented in Fig. 3, Pressure had a slight positive relation with CO_2_ uptake capability (*r* = 0.37), and a mildly negative relation with temperature (*r* = − 0.24). However, based on the total adsorption data, the CO_2_ adsorption capacity was determined to have a limited association with the characteristics of porous GOs. The adsorption capacity was positively and weakly related to total pore volume (*r* = 0.2); on the other hand, the adsorption capacity was positively and highly related to surface area (*r* = 0.55), which was consistent with previous studies finding out that higher surface area resulted in higher adsorption capacity.

**Figure 4:**
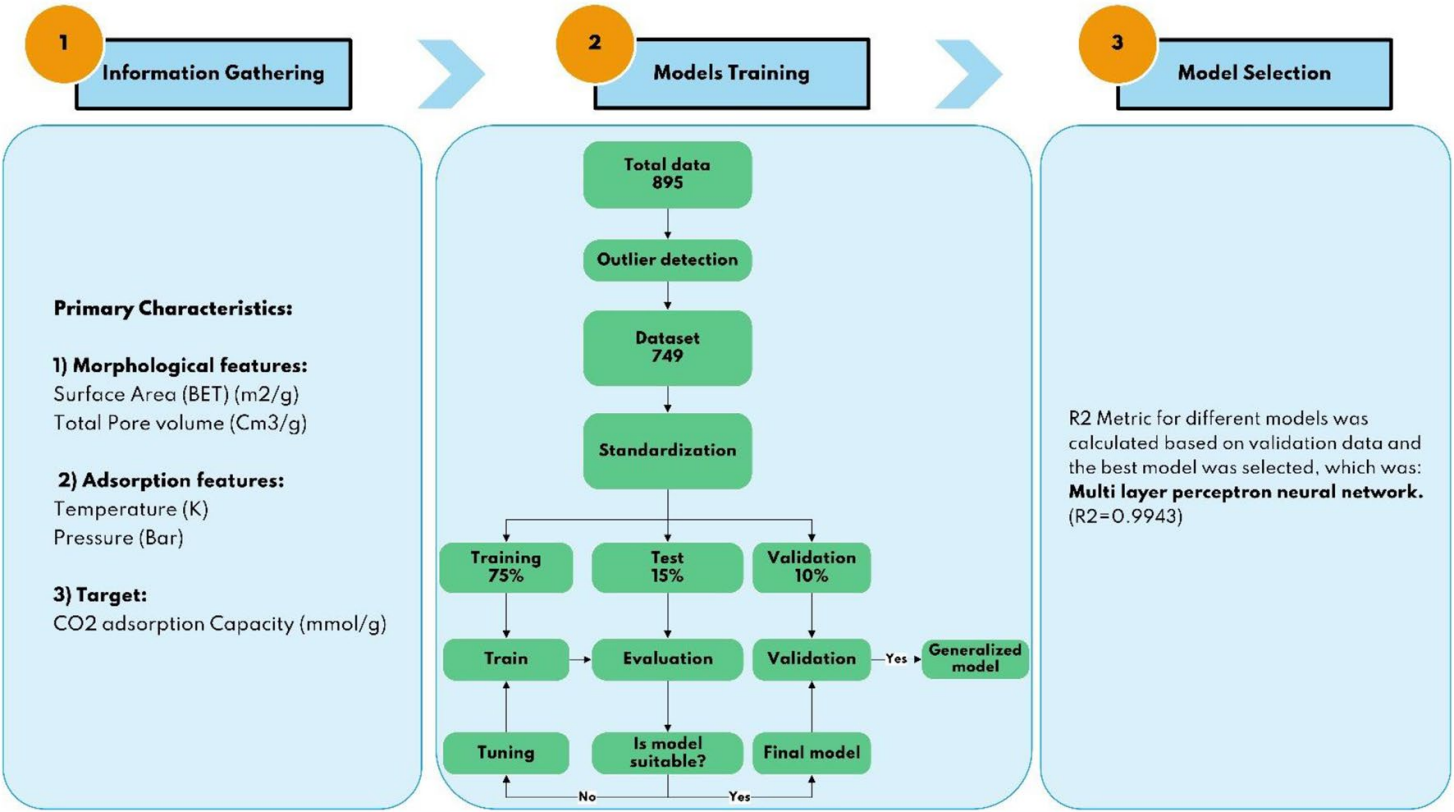
Procedure and algorithm diagram of the current machine learning-based modelling.

The resulting data were divided into 3 parts: 75% of the data was considered as training data, from the remaining 25%, 60% was defined as test data for models hyperparameter optimization, and remaining 40% was defined as validation data (unseen data). Machine learning algorithms do not work well when numerical features have exceptionally distinctive scales, so feature scaling is one of the most critical changes to be made to the data. There are two common ways to urge all properties to have the same scale including normalization and standardization. Unlike normalization, standardization does not bound values to a particular extent. In any case, standardization is much less influenced by outliers^30^. For this purpose, the standardScaler class from preprocessing module of the scikit-learn (sklearn) was used, which its formula is presented here.

1 $$\mathrm{Z}=\frac{\mathrm{x}-\mathrm{u}}{\mathrm{s}}$$

where *u* is the average value of the training samples and *s* is each training sample's standard deviation. Figure 4, shows the general procedure of data gathering and data classification for training the mentioned machine learning models aim to achieve the best model.

## Modeling methods

### Model selection

Various machine learning methods or models may be used to solve numerous classification, clustering, and regression problems. The current challenge is that whichever model and hyperparameter combinations would function better upon the particular dataset. The optimization algorithm in this scenario contains several learning algorithms (models) and hyperparameters. One needs to produce many hyperparameter combinations to maximize predictive accuracy and obtain the optimal collection of hyperparameters. Next, the one that yields the best predictive precision may be achieved by exploring hyperparameter combinations. Grid search may be employed to detect the optimal collection of hyperparameters by searching across all possible permutations. The sklearn library's "GridSearchCV" function can be utilized to connect linear search through hyperparameters. The sets of all hyperparameters to be adjusted are handed to GridSearchCV. The GridSearchCV develops a design based on the optimum hyperparameter combination for the incoming and outgoing parameters^31^. In this study, seven mentioned models are used, which their brief explanations are presented first. The models are Random forest, support vector machine (SVM), gradient boosting, extra trees, extreme gradient boosting (XGB), and ANN (MLP, RBF), respectively.

#### Isolation forest

This model can be a proficient calculation for outlier detection. The calculation builds an Irregular Forest in which each Chosen Tree is developed arbitrarily; at each node, it picks a feature at random; at that point, it picks an arbitrary limit value (between the minimum and maximum values) to part the dataset in two sections. The dataset slowly gets chopped into pieces this way until all occurrences are separated from one another. Inconsistencies are ordinarily distant from other instances, so on regular (overall the Chosen Trees), they tend to urge separated in fewer steps than typical instances.

#### Support vector machine (SVM) regression

SVM is a training machine learning technique that may be utilized for classification and regression tasks. In contrast to many ML algorithms, during which the goal is to minimize the cost function. The primary goal of SVM seems to be maximizing the margin among support vectors via a separating hyperplane^32^. It covers not only linear and nonlinear classification but also covers linear and nonlinear regression. The secret to using SVMs for regression rather than classification is to reverse the goal. In this work, to do SVM Regression, the SVR class from the SVM module from scikit-learn API was used.

#### Random forest

**Figure 5:**
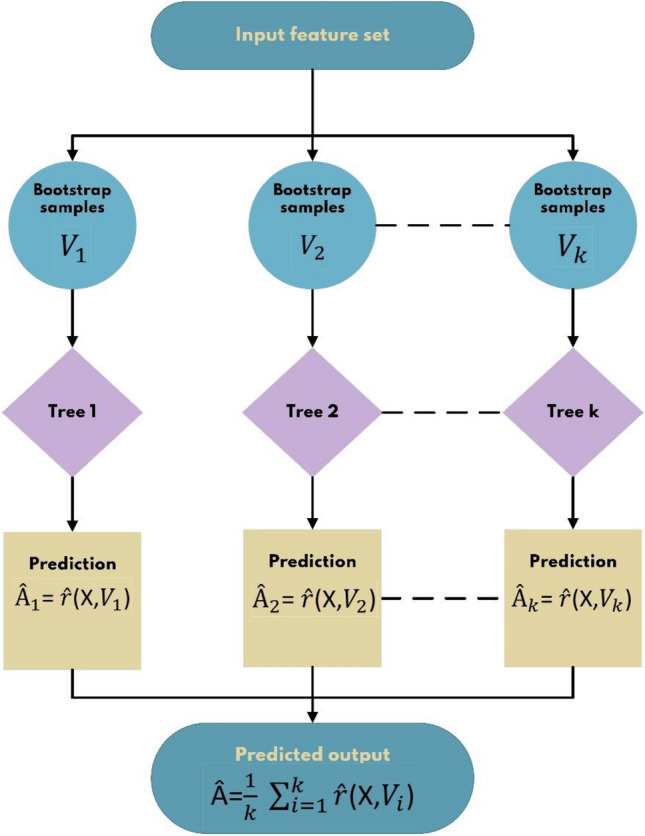
Schematic diagram of random forest procedure.

Random Forest is a simple machine learning algorithm that typically generates excellent results even when its meta-parameters are not adjusted. This algorithm is among the most extensively employed ML algorithms for both "Regression" and "Classification" because of its simplicity and applicability. The random forest algorithm starts by dividing the input features into subsets that form a tree; then, a proper fitting function is developed for each decision tree that works on the random features picked. A random forest model is built at the end of the training procedure. It is worth noting that every tree is built from randomly chosen input vectors during the training process, namely "random" forest^33^. For implementing this model, the RandomForestRegressor class from the ensemble module in the scikit-learn API was employed. Figure 5 illustrates a schematic of how the random forest model works.

In Fig. 3, ${\hat{\text{r}}}\left( {X,V} \right)$ is the representative tree at the end of the training phase, *X* is the set of input feature vectors, *T* is the collective set indicating the input–output pair V_i_ = (*x*_1_, *y*_1_), (*x*_2_, *y*_2_),…(*x*_*n*_, *y*_*n*_), and *k* is the number of trees.

#### Extra trees regressor

Extra trees are a supervised machine learning technique comparable to the random forest and can be harnessed for regression and classification. In a Random Forest, just a random subset of the features is considered for splitting at every node. Instead of searching for the best possible thresholds, trees can be made even more random by applying random thresholds for every feature. A forest of such highly random trees is named an extremely randomized trees ensemble. Such a strategy trades more bias for less variance. Also, it makes extra-trees significantly quicker to train than standard Random Forests since one of the most time-consuming aspects of tree growth is detecting the optimum threshold for every feature at each node^34^.

#### Gradient boosting

**Figure 6:**
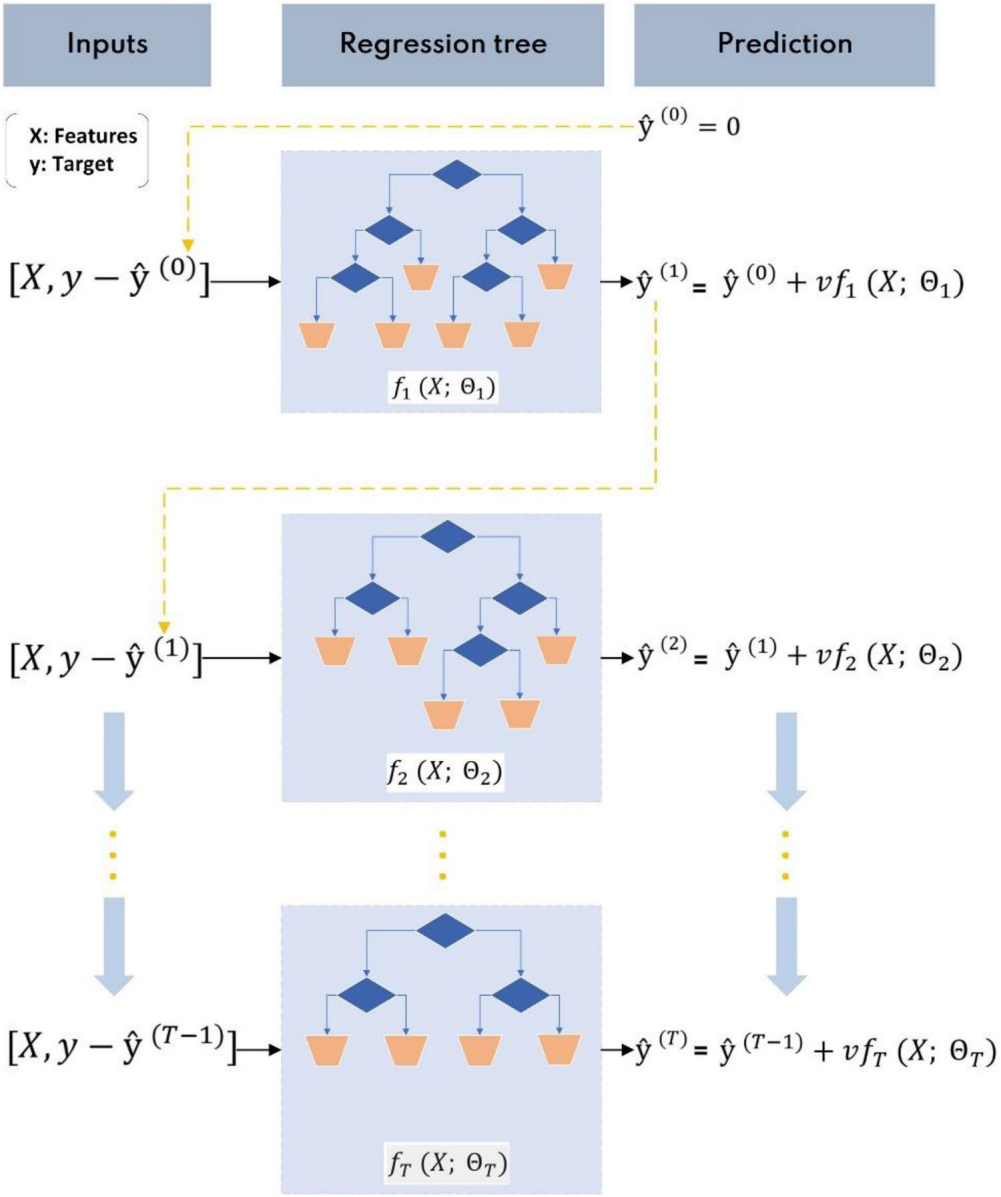
Schematic diagram of gradient boosting procedure.

Gradient boosting is an ensemble supervised ML method that may be utilized for regression and classification. The term "ensemble" refers to methods, like random forest, extra trees, gradient boosting, that builds an ultimate model according to various individual models. Gradient boosting trains several models sequentially by assigning greater weights to examples with incorrect predictions. As a result, tough instances are the focus of training. Gradient boosting is used in sequential model training to gradually reduce a loss function. This function will be minimized in the similar way as an ANN model^35^. GBR provides several advantages, remarkedly strong prediction accuracy and stable output. The additive training mechanism of the boosted model may be represented in a forward linear way as:

2 $$\begin{aligned} & \hat{y}^{\left( 0 \right)} = 0 \\ & \hat{y}^{\left( 1 \right)} = vf_{1} \left( {x;\Theta_{1} } \right) = \hat{y}^{\left( 0 \right)} + vf_{1} \left( {x;\Theta_{1} } \right) \\ & \hat{y}^{\left( 2 \right)} = v\mathop \sum \limits_{j = 1}^{2} f_{j} \left( {x;\Theta_{j} } \right) = \hat{y}^{\left( 1 \right)} + vf_{2} \left( {x;\Theta_{2} } \right) \\ & \ldots \\ & \hat{y}^{\left( T \right)} = v\mathop \sum \limits_{j = 1}^{T} f_{j} \left( {x;\Theta_{j} } \right) = \hat{y}^{{\left( {T - 1} \right)}} + vf_{T} \left( {x;\Theta_{T} } \right) \\ \end{aligned}$$

where *T* is the number of RTs for boosting; *Θ*_*j*_ is the structure of the *j*th RT; *ν* is the shrinkage parameter (distinguished by the learning rate that satisfies 0 < *ν* < 1 for shrinking the contribution of RTs); ${\widehat{y}}^{(j)}$ is the estimation of target variable by first *j* RTs; and ${f}_{j}$ is the output of the *j*th RT without shrinkage, which employs predictor variables *x* to approximate $y-{\widehat{y}}^{(j-1)}$ (i.e., residuals) with tree structure *Θj*. As the number of RTs grows, the residuals will normally decrease. Figure 6 depicts a schematic diagram of the Gradient boosting procedure for illustrative purposes^36^.

#### Extreme gradient boosting (XGB)

Tianqi Chen invented extreme gradient boosting, often called XGBoost, as a ML method that may be utilized for regression and classification. XGBoost is a gradient boosting approach that distinguishes from a gradient boosting model in multiple ways: (1) because of the multithreading of tree structures, XGBoost is generally quicker than gradient boosting, (2) because it can accept incomplete data inside a collected data, data preprocessing takes less time^37^. The XGBRegressor class from the xgboost package was used to implement this model.

#### ANN-MLP

In the early 1940s, the network technique was utilized to assess and analyze data for many themes, and the ANN structure was applied. Currently, scientists are working to improve understanding of how the human brain works to create the next generation of neuroscientific machine learning^38^. One of the benefits of the neural network is that it needs less time to solve complicated problems. If there is no specific relation between the data, ANNs, as patterned after the human biological brain, are harnessed to discover one. The neural network has the following characteristics: parallel computing (top intensity), nonlinear calculations, generality, output and input data interchange, adaptability, large data response, error tolerance, and training^39^. The neural network approach describes as human nerve anatomy. McCulloch and Pitts invented the ANN based on the activity of actual elements of the brain. The analysis process in neural networks is similar to the operations of neurons in human brains^40^. The functioning of neurons in the human brain is quantitatively represented in ANNs. The terms neural networks (NNs) and ANNs will be used equally henceforth. NNs have two potential applications: Discovering a relationship among a group of quantitative inputs (features) and outputs (target) and clustering. In general, NNs are made up of a set of "Neurons" arranged in a layered architecture. Every input and output variable may correspond to a node, which functions similarly to a real neuron. Nodes are organized into layers in which input and output layers are linked. The number of hidden layers and the number of nodes per each that link the input to the output layer are specified by the architecture of NNs. Weights (*w*_*ij*_) indicate the link among each of the two nodes, where *i* and *j* demonstrate nodes in the source and destination nodes, respectively^41^. The ANN approach is also one of the most extensively utilized techniques in nonlinear applications. This method's excellent properties include nonlinearity, classification, identification, data analysis, and optimization. In the NN approach, the network design is taught based on experimental data, and all parameters in the network model are optimized to achieve the best result. The target in ANN is to obtain the proper weights (*w*) for a specific function (*f*). Every input (*x*_*i*_) is multiplied by the relavant weight, all quantities are added together, and then the threshold or bias quantity (*b*) is added to the sum of the quantities. The equation below represents this approach for input data:

3 $$sum=\left(\sum \limits_{i=1}^{N}{\omega }_{i}{x}_{i}\right)+b$$

The output quantities, *y*, are created by feeding the data into a transfer function, *f*, as given in Eq. (4).

4 $$y=f(sum)$$

The common transfer functions are step, Relu, LeakyRELU, hyperbolic tangent, and sigmoid (S shape).

**Figure 7:**
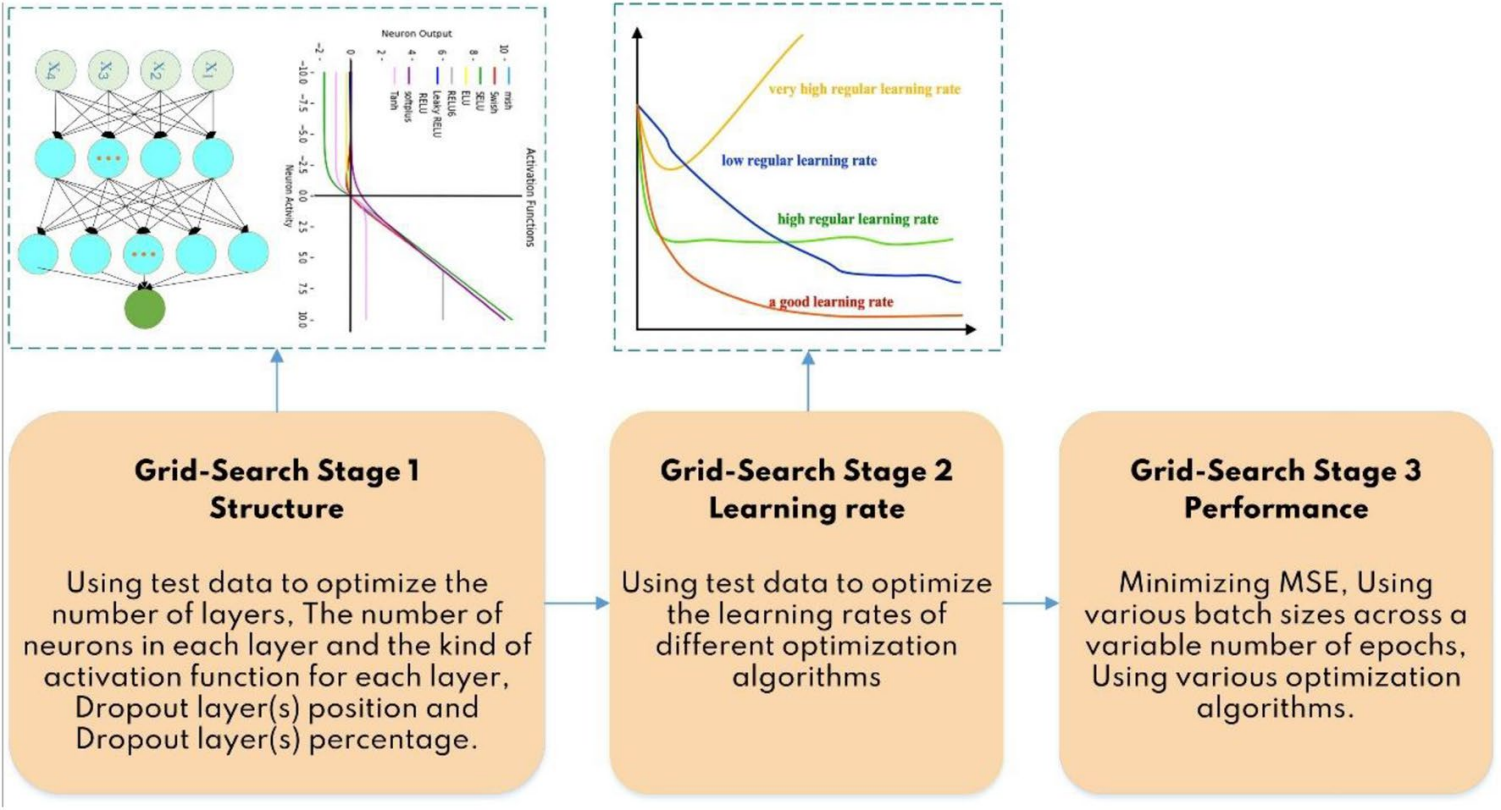
Different stages for optimizing the ANN models.

Optimization algorithms or optimizers are critical components in improving the performance of a NN They conventionally adjust the hyperparameters of a model based on its design. Hyperparameters that impact an optimizer's behavior, such as learning rate, control its update rule, determining the optimizer. The integration of hyperparameters and update rule separates any two optimizers. An optimizer must adjust the weights and learning rate of the model's nodes throughout the training phase to minimize the loss function. To summarize, the primary aim of an optimizer is to minimize training error^42^. The optimization procedure of the best ANN algorithm is summarized in Fig. 7.

Overfitting and extended training times are two significant difficulties in multi-layered neural network learning, especially deep learning. Overfitting occurs when a model conducts properly on training data but badly on test data; in other words, the model has low training error but high test error. Regularization is a collection of approaches for decreasing overfitting. Dropout advocated randomly changing the network architecture when overfitting in deep learning to lessen the risks that the learnt weight values are excessively customized to the underlying training data and consequently cannot be generalized properly to test. Dropout simulates model ensembling without the need for several networks^43^.

Adam optimizer was utilized to solve the network, an algorithm for first-order gradient-based optimization of stochastic objective functions according to adaptive predictions of lower-order moments. This method is simple to advance, computationally effective, needs minimal memory, invariant to gradient diagonal rescaling, and is ideally suitable for issues with immense amounts of data and/or parameters. The hyperparameters have straightforward interpretations and need a slight adjustment in most cases^44^.

#### Radial based function (RBF)

The radial based function (RBF) neural network is a feedforward network with an individual hidden layer; also, Broomdhead and Lowe suggested this network for the first time^45^. The solution of an over-specified set of linear correlations can be solved using some highly stable approaches during the training of RBF networks with pre-determined nonlinearities. The RBF networks have a solid theoretical foundation since they are closely related to the well-studied field of linear models' regularization theory^46^. The data from the input layers are gathered from the hidden layer and moved forward the Gaussian transfer function, converting the data into nonlinear functions. The RBF algorithm utilizes nonlinear transfer functions to link the hidden and input layers. The geometrical dimension-based distance between the weights and the output vector is determined by the individual hidden neurons in the network. Equation (6) presents the combiners-based RBF algorithm network output layer in its linear form:

6 $$f(x)=\sum \limits_{i=1}^{N}{w}_{ij}G\left(\Vert x-{c}_{i}\Vert *b\right)$$

where* N* is the number of training data sets, *W*_*ij*_ is the weight attributed to every hidden neuron, *x* is the input vector, *c*_*i*_ is the center points, and *b* is the bias. A Gaussian equation, Eq. (7) can be employed to detect the centralized solution from the hidden point, as follows:

7 $$G(\Vert x-{c}_{i}\Vert *b)=\mathit{exp}\left((-\frac{1}{2{\sigma }_{i}^{2}}(\Vert x-{c}_{i}\Vert *b{)}^{2}\right)$$

The Gaussian function's spread is *σ*_*i*_. This equation is the range of $\Vert x-{c}_{i}\Vert$ within the input domain to which the RBF neuron can respond. The procedure of choosing neurons in the RBF network is typically according to trial and error, thus the algorithm begins with a considerable number of neurons in the single hidden layer and then is conducted to decrease the number of neurons as much as the minimum *MSE*.

In this work, Rmsprop optimizer was harnessed to train the network. RMSprop and Adadelta entered the scene concurrently but independently, intending to cope with Adagrad's diminishing learning rates. RMSprop is a gradient-based optimizer that, rather than treating the learning rate as a hyperparameter, uses an adaptive learning rate that varies over time^47^.

### Error metric

The performance of the models is compared by the following metrics (*RMSE*, *R*^2^, *MSE*, *MAE*), and ultimately, the criterion *R*^2^ is considered to select the best model.

*Mean absolute error (MAE)* It is just the mean of the absolute difference between the estimated and actual data, which can be calculated as follows:

8 $$MAE= \frac{1}{n}{\sum }_{i=1}^{n}\left|{y}_{i}-{\widehat{y}}_{i}\right|$$

*Mean squared error (MSE)* As the title implies, it is the mean of the squared errors. *MSE* can also be taken into account as a loss function that must be decreased. It is often utilized in real-world machine learning applications because greater errors are penalized more when employing *MSE* as the objective function than when using *MAE*^35^.

9 $$MSE=\frac{1}{n}\sum_{i=1}^{n}{({y}_{i}-{\widehat{y}}_{i})}^{2}$$

*Root Mean Square Error (RMSE) RMSE* is the square root of *MSE*^35^.

10 $$RMSE=\sqrt{\frac{1}{n}\sum_{i=1}^{n}{({y}_{i}-{\widehat{y}}_{i})}^{2}}$$

*Coefficient of determination (R*^*2*^*)* It assesses the model's fitness to the liable, scientific results. The nearer the coefficient of determination (*R*^2^) is to 1, the higher the predictions fit the experimental data. *R*^2^ is calculated as follows^48^:

11 $${R}^{2}=\frac{{\sum }_{i=1}^{n}{\left({Y}_{predicted}-{Y}_{actual}\right)}^{2}}{{\sum }_{i=1}^{n}{\left({Y}_{predicted}-{Y}_{mean}\right)}^{2}}$$

where *Y*_*mean*_ is the mean of the actual quantities.

## Results and discussion

### Hyperparameters of each model

*SVM* The mentioned hyperparameters are properly considered when the optimization of SVM is carried out: (*C*, gamma, kernel, and epsilon), and the optimal values are 2500 for *C*, the gamma is scale, the kernel is rbf, and epsilon is 0.0075.

*Random forest* To optimize the random forest, the following hyperparameters are considered: (n_estimators, min_samples_leaf, and min_samples_split), where the optimal values are 700, 2, and 1 for n_estimators, min_samples_split, and min_samples_leaf, respectively.

*ExtratreesRegressor* To tune ExtraTreesRegressor, the following hyperparameters are considered: (max_features, n_estimators, min_samples_split, min_samples_leaf, and max_depth), the optimal values are 3372 for n_estimators, and the criterion is squared_error, min_samples_split = 2, and min_samples_leaf = 1.

*Gradient boosting* The following hyperparameters are considered to optimize the Gradient BoostingRegressor. (n_estimators, learning_rate, criterion) where the optimal values are 900 for n_estimators, the learning_rate = 0.4 and the criterion is friedman_ *MSE*.

*Extreme gradient boosting* To optimize XGBRegressor, the following hyperparameters are considered: (n_estimators, learning_rate, reg_alpha, booster, gamma, and reg_lambda). The optimal values are as follows: n_estimators = 2800, learning_rate = 0.2, reg_alpha = 0.1, booster = “dart”, gamma = 0.0001, and reg_lambda = 0.92.

**Figure 8:**
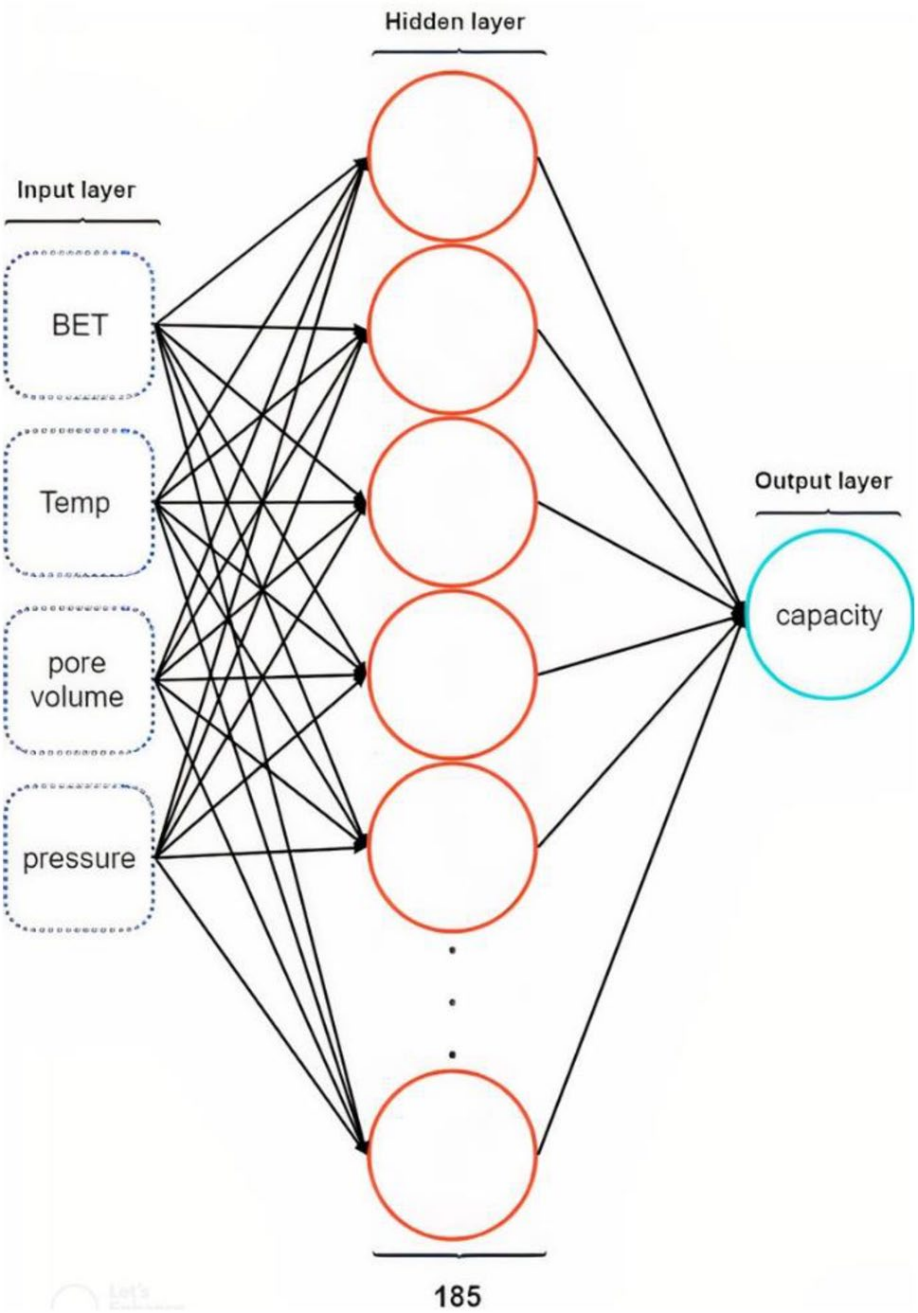
Schematic diagram of RBFNN model used for optimizing.

*RBFNN* The RBF network training was conducted through optimization of the network characteristic such as, the number of neurons, the number of epochs, the used optimizer, the learning rate, and the batch_size to achieve the best result on the test data. This model was coded using the TensorFlow API. The tuned parameters are the number of neurons = 185, the optimizer is RMSprop, the lerning_rate = 0.003, epochs = 4500, and the batch_size = 32. Figure 8 depicts a schematic of this type of neural network, whereas its learning curve will be shown in Fig. 10.

**Figure 9:**
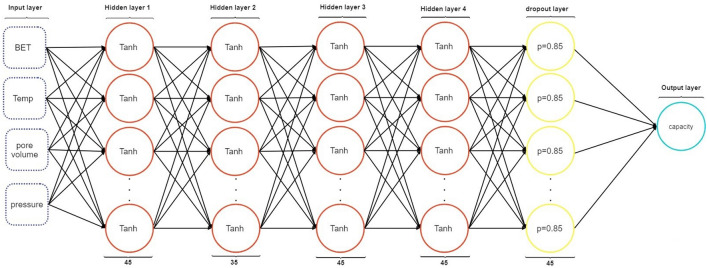
Schematic diagram of MLP network.

*MLP* The MLP network architecture, which includes the number of neural network layers, number of neurons per layer, activation function per layer, dropout layer percentage, dropout layer(s) position, number of epochs, used optimizer, learning rate, The *β*_1_ and *β*_2_ parameters for adam optimizer, and batch_Size were considered to achieve best results on test data. The neural network architecture used can be seen in Fig. 9.

**Figure 10:**
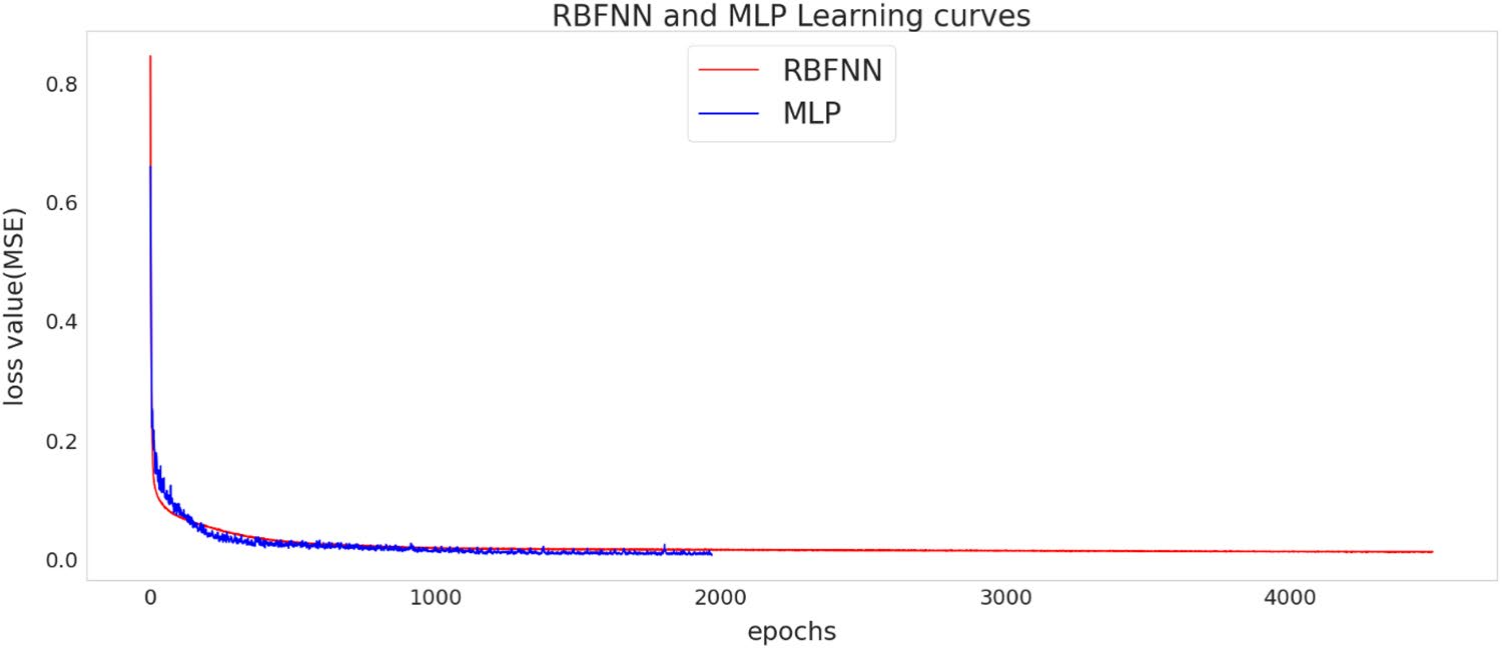
The learning curve of ANN (MLP and RBF) models.

The optimal hyperparameters of MLP network are as follows: optimizer = adam, the learning_rate = 0.003, *β*_1_ = 0.9, *β*_1_ = 0.999, epochs = 1971 and batch size = 32. For applying this neural network, the Dense model of the Keras module in the TensorFlow API has been employed. Figure 10 shows the learning rate of the optimal architecture of the MLP and RBF networks.

The *MSE* and *R*^2^ were utilized as assessment parameters to link the model outputs to the validation data. Analytical criteria, such as *MAE*, *RMSE*, *R*^2^, and *MSE* as reported in the previous equations, are employed to evaluate the model’s performance. In the last stage, the optimum model is selected. The results are shown in Table 2. The ANN-MLP was the best-fitted model for predicting the experimental results. This model has a *MSE* of 0.024 and a maximum *R*^2^ value of 0.9943, while the RBF model has a *MSE* of 0.103 and a maximum *R*^2^ value of 0.9747. According to the *R*^2^ and *MSE* values which reported in Table 2, we can choose MLP as the best model algorithm among these seven models. In order to further testing of the optimal network performance, some of experimental values of CO_2_ adsorption which results from the papers were selected randomly and compared with the model predicted values. The results of comparison are presented in the next section.

**Table 2:** Analytical criteria for models comparison.

Model	*R*^2^	*MAE*	*MSE*	*RMSE*
ANN	0.994	0.097	0.024	0.153
ExtraTreesRegressor	0.989	0.109	0.043	0.207
GradientBoostingRegressor	0.987	0.101	0.055	0.234
RandomForestRegressor	0.981	0.175	0.078	0.280
XGBRegressor	0.979	0.139	0.086	0.293
RBFNN	0.975	0.189	0.103	0.321
SVR	0.965	0.188	0.141	0.375

### Comparison between experimental datas and predictions

**Figure 11:**
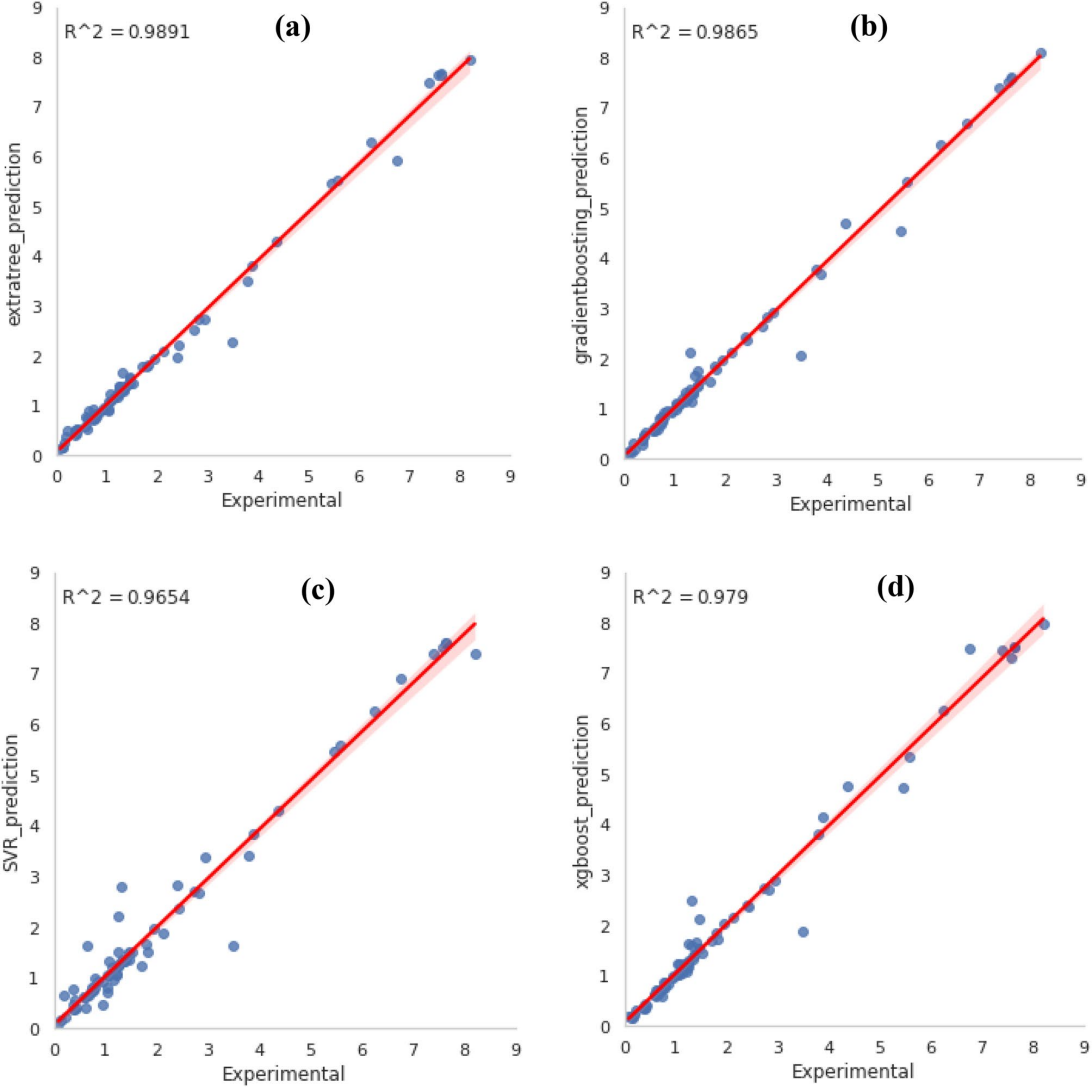

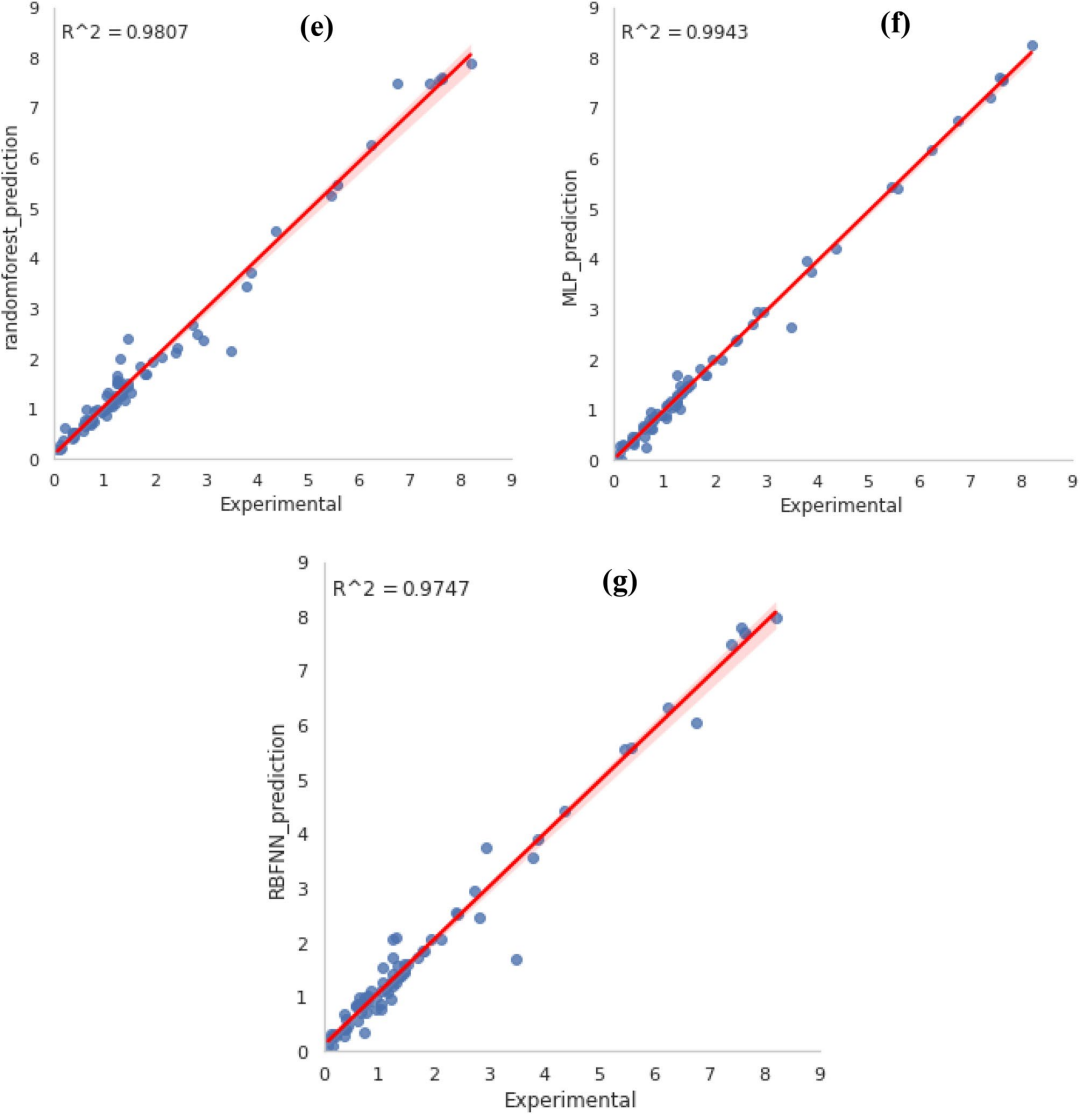
CO_2_ adsorption experimental versus predicted data using the models: (**a**) Extratree, (**b**) Gradientboosting, (**c**) SVM, (**d**) Extragradient boosting, (**e**) Randomforest, (**f**) ANN-MLP, and (**g**) ANN-RBF.

The specified hyperparameters were employed to retrain the models with training datasets (75%), which were then verified by validation data (10%) in each case. The graph compares estimated CO_2_ adsorption capacity to experimental quantities of test groups depicted in Fig. 11. The high *R*^2^ (0.9943) and low *MSE* (0.024) values confirmed that the ANN-MLP model is suitable to estimate the CO_2_ capture capacity of GOs based on their structural characteristics and adsorption conditions. The precise ML model, not as it were, may foresee the CO_2_ adsorption capacity under various adsorption conditions for modern new GOs with diverse structures. Moreover, it may overcome a few lacks of conventional adsorption isotherm models (for example, Langmuir model). The reasons are (1) ML models are not constrained by type of adsorbents and adsorption conditions. In contrast, the model parameters of conventional isotherm models are not applicable to utilizing diverse temperatures or adsorbents with various morphological features. (2) With ML models, experimental data was directly used without making verifiable assumptions like Langmuir's monolayer adsorption^49^. Thus, ML models created in this work could decrease time-consuming and costly investigational screening tests for various adsorbents utilized in diverse scenarios, thereby facilitating cost-effective and cleaner generation for green supportability. Figure 11 suggests an elevated level of accuracy in the organization between the ANN-MLP outputs and the CO_2_ adsorption data. The experimental data provided here were also frequently agreed with the model predictions (Fig. 11). With an *R*^2^ quantity of 0.9943 and an *MSE* quantity of 0.024, the ANN-MLP model achieved the most accurate result, showing that it correctly estimates the experimental data.

To check the accuracy of the obtained models, seven papers were selected randomly among the considered ones. In Table 3, the amount of experimental absorption given in these papers, The calculated value is determined according to the operating conditions with each model separately. Also, the ANN-MLP model indicated the most accurate prediction of CO_2_ adsorption in most cases reported in Table 3 among all models.

**Table 3:** Calculation of adsorption paraemters by models by fitting the experimental data.

References	Adsorbent name	*BET* Surface area (m^2^/g)	Temperature (K)	Total pore volume (cm^3^/g)	Pressure (bar)	Actual value	SVR	Random forest	Extra tree	Gradient Boosting	Xgboost	ANN-MLP	RBFNN
^50^	GEPM-1	253	273	0.7	0.24	1.52	1.53	1.32	1.44	1.57	1.44	1.52	1.60
^51^	TiO_2_ /GO-0.2	87.77	273	0.3	0.9	1.45	1.50	1.52	1.52	1.48	1.54	1.45	1.49
^52^	CG-3	1470	273	0.61	0.52	5.45	5.45	5.25	5.47	4.54	4.73	5.44	5.57
^22^	aPPy/GO-20	2270	273	1.1	1	6.75	6.91	7.42	5.97	6.70	7.48	6.74	6.03
^51^	TiO_2_ /GO-0.1	99.54	273	0.38	0.21	0.79	0.80	0.75	0.79	0.84	0.86	0.80	0.91
^53^	CTS/GO-5	241	298	0.89	0.7	2.43	2.36	2.27	2.22	2.38	2.37	2.41	2.52
^54^	CuBTC/GO-5	1637	273	0.67	0.1	1.36	1.34	1.37	1.40	1.34	1.41	1.38	1.40

**Figure 12:**
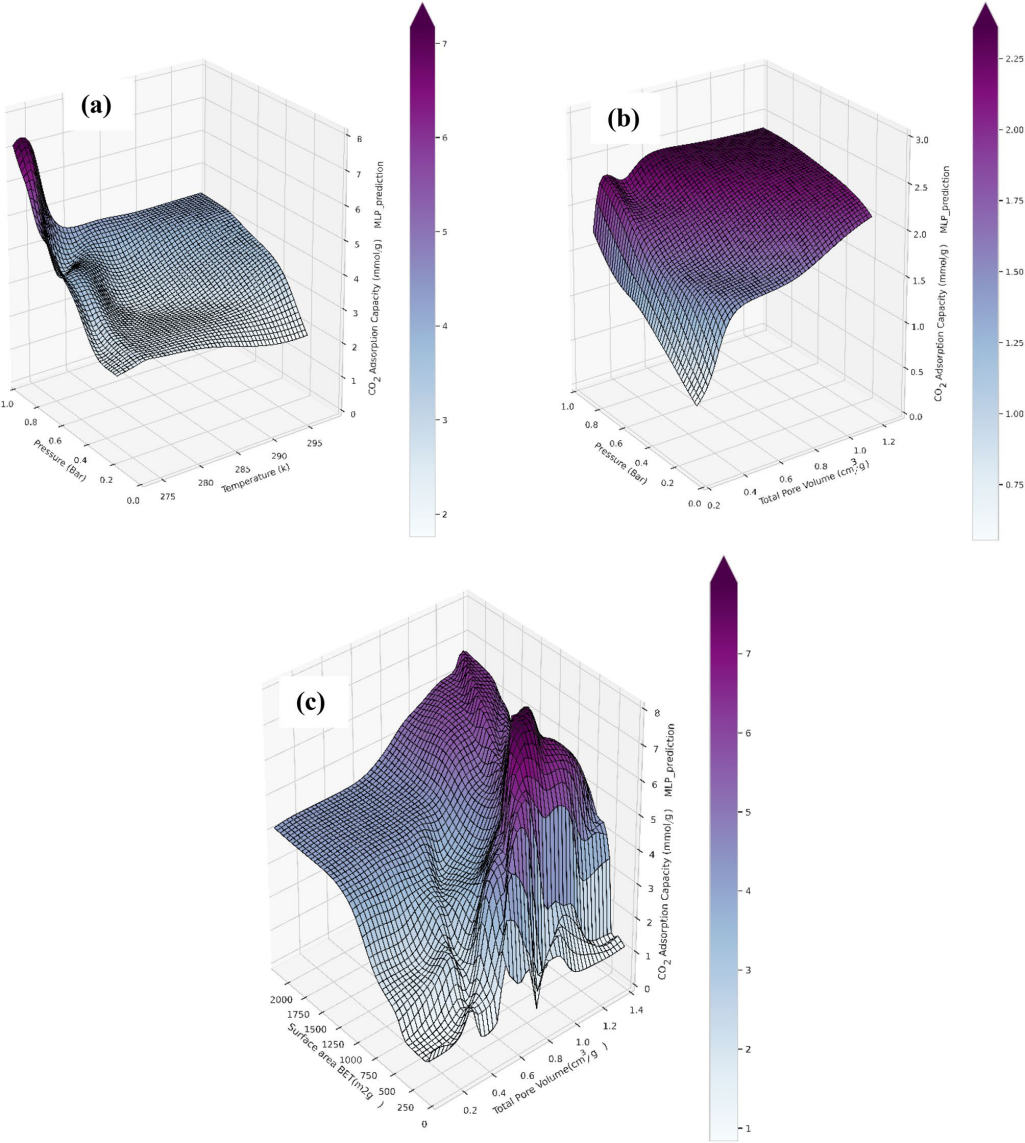
3D response surface plots generated by ANN-MLP model: (**a**) temperature verus pressureat *BET* = 1500 and *pore volume* = 0.4 (**b**) pressure versus pore volume at *T* = 273 K and *BET* = 99.54 (**c**) *BET* versus pore volume at *T* = 303 K and *P* = 1 bar.

According to the reported data, ANN-MLP is the best algorithm for predicting experimental data related to CO_2_ adsorption. The network's training algorithm seeks to reduce the mean of the overall inaccuracy. Thus, the ANN-MLP model was used for obtaining three dimensional graphs which show the relationship between structural parameters or adsorption conditions and CO_2_ adsorption capacity. Figure 12 illustrates the ANN-MLP forecasting model's 3D curves. The curves were gathered in order to understand better the effects of textural factors (*BET* surface area and total pore volume) and operational conditions (temperature and pressure) on CO_2_ adsorption capacity. According to Fig. 12a, At a constant temperature (273 K), the CO_2_ uptake increases with enhancing pressure, it can be related to improving the mass transfer driving force and enhancement of diffusion of the CO_2_ molecules inside tha adsorbent cavities. At constant pressures, temperature increases from 273 to 298 K slightly decreases The CO_2_ adsorption capacity, it can be related to physically nature of CO_2_ adsorption by graphene oxide. Although graphene oxide is a porous media with various types of functional group, but it should be considered that lack of electron donor group (Lewis base) such as amine or amides causes the CO_2_ adsorption process occurs physically through weak dipole-quadropole interaction between CO_2_ molecules and adsorbent surface^55^. Figure 12b displays the effect of pressure and total pore volume on CO_2_ adsorption capacity. At constant pressure, the adsorption capacity increases with enhancing the total pore volume. When the pressure is low (0.2 bar), the rate of capacity increment is considerable in the range of pore volume between 0.1 and 0.5 cm^3^/g. After this range, the rate of capacity enhancement is not remarkable. The best condition for CO_2_ adsorption is when the pressure and total pore volume are high. Figure 12c shows the effect of surface area and pore volume on the adsorption capacity. According to this figure, at a constant pore volume, the relationship between CO_2_ capture capacity and *BET* surface area is positive and remarkable, meaning that the CO_2_ uptake increases by enhancing *BET* surface area. For surface areas < 1500 m^2^/g, increasing pore volume would not improve the adsorption, as other important factors have an enormous influence on the quantity of adsorption; however, when *BET* > 1500 m^2^/g at a constant surface area, the adsorption capacity increases slightly with increasing the pore volume.

**Figure 13:**
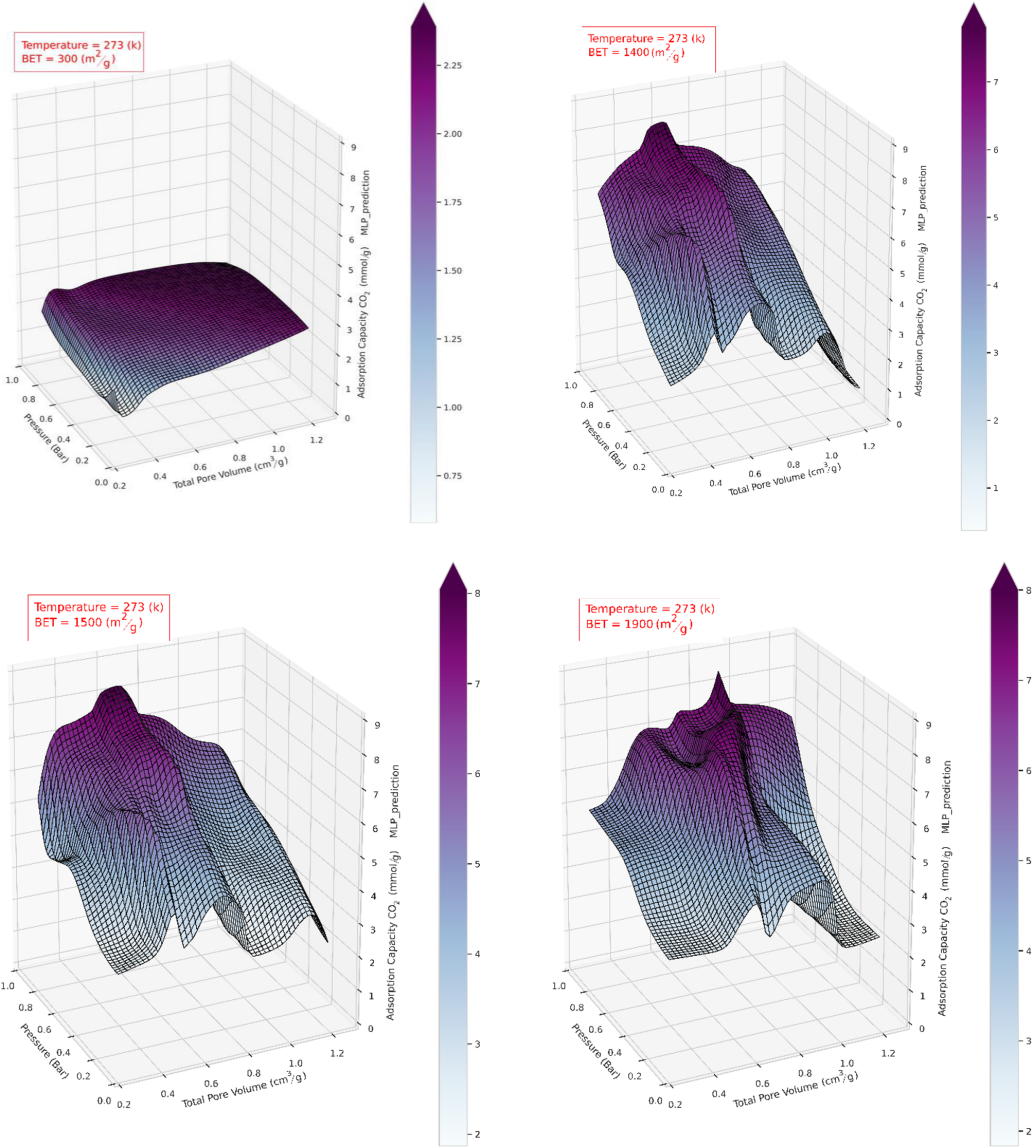
3D response surface plots generated by ANN-MLP model. Total pore volume and pressure versus adsoption capacaity at various temperatures and *BET*s.

As it can be seen in Fig. 13, *BET* surface area has a considerable impact on the CO_2_ adsorption capacity, and by increasing *BET* area from 300 to 1400 m^2^/g, the adsorption capacity has raised upon three times, but more importantly, at high values of the *BET BET* area, increment of the pore volume does not necessarily increase the CO_2_ uptake capacity. After a specific value of the pore volume, usually around 0.8 (cm^3^/gr), the increment of pore volume at a constant *BET* area decreases the adsorption capacity, which can be related to the reduction in ratio of the volume of mesopores to the total pore volume and the pore diameter incrreasing, which reduces the adsorption capacity.

This project proposes a sound and efficient methodology to predict CO_2_ adsorption and optimize CO_2_ adsorption linked to various GO-based adsorbents with the help of seven different algorithms. Also, it provided confidence in the ANN designs as predictive deep learning utilizing CO_2_ adsorption and properties of GO-based adsorbents through producing more reliable estimations for CO_2_ uptake in industrial operations.

## Conclusions

This study studied several GO-based adsorbents to establish a machine learning prediction for CO_2_ adsorption. A massive amount of data was collected from 19 articles (895). Several models have been employed to predict CO_2_ adsorption capacity. Among them, the ANN-MLP model demonstrated the best estimation with *R*^2^ of 0.9943 and *MSE* of 0.024. To investigate the effect of temperature, pressure, surface area of GO, and total pore volume on CO_2_, three dimensional surfaces were reported, also the MLP network weight and biases matrix were reported for further process design applications. The findings revealed that pressure and surface area were the most influential factors in CO_2_ adsorption capacity. Textural characteristics (surface area and total pore volume) were more important than chemical compositions of adsorbents in their CO_2_ adsorption capacity at different temperatures and pressures. If additional significant parameters are incorporated with sufficient data, CO_2_ adsorption models can be more comprehensive and reliable. In the future, interaction software might be produced to allow the straight identification of suitable adsorbents for diverse CO_2_ adsorption requirements in numerous applications.

## Supplementary Information

Supplementary Information.

## Data Availability

Data are available [from Farnoush Fathal] with the permission of [Alireza Hemmati]. The data that support the findings of this study are available from the corresponding author, [Alireza Hemmati], upon reasonable request.

## List of symbols

*b*: Bias (–)
*C*_*i*_: Center points (–)
*f*: Activation function
*G*: Gaussian function
*i*: Number of neurons in each hidden layer
*N*: Number of data for training (–)
*n*: Neurons
*R*^2^: Coefficient of determination
*W*: Weight matrix (–)
*W*_*ij*_: Weight-related each hidden neuron (–)
*Exp*: Experimental value (–)
*X*: Input variable (–)
*x*_*i*_: Input examples (attributes)
*Y*: Output vector (–)
*y*_*j*_: Target output

## Greek symbols

$\sigma$: Width of radial basis function (RBF) kernel (–)
*σ*_*i*_: Spread of Gaussian function (–)

## Abbreviations

ANN: Artificial neural network
MSE: Mean square error
MAE: Mean absolute error
RMSE: Root mean square error

## Terminology

Outlier: A point that's endlessly distinctive from other focuses in a dataset
Outlier detection: The method of finding outliers in a dataset
Neurons: The basic units of the large neural network
Bias: A constant which helps the model in a way that it can best fitted for the fit data
Activation function: A mathematical function between the input feeding current neuron and its output going to the next layer
Weight: Represents the importance and strengths of the feature/input to the Neurons
Epoch: In the training process, the inputs entering in each training step and giving an output that is compared with the target to calculate an error. With this process, weights and biases are calculated and modified in each epoch
