# Supplementary material for: Intelligent prediction models based on machine learning for CO2 capture performance by graphene oxide-based adsorbents
Source: Sci Rep. 2022 Dec 13;12:21507. doi: 10.1038/s41598-022-26138-6 (PMC9747901; doi:10.1038/s41598-022-26138-6)
Supplement: Supplementary file 1 — Supplementary Information. [file 41598_2022_26138_MOESM1_ESM.docx]

**Table A1.** Experimental range and results of CO_2_ capture process by GO-based adsorbents obtained from the literature.

| **Name of Adsorbent** | **GO Loading %** | **Surface area (m^2^/g)** | **Total Pore Volume (cm^3^/g)** | **T (K)** | **P (bar)** | **CO_2_ Capture Capacity (mmol/g)** | **Ref.** |
| --- | --- | --- | --- | --- | --- | --- | --- |
| Chitosan-GO | CTS/GO-1 | 182 | 0.76 | 298 | 0.11 | 1.12.16 | 53 |
| Chitosan-GO | CTS/GO-3 | 205 | 0.78 | 298 | 0.11 | 1.172.53 |  |
| Chitosan-GO | CTS/GO-5 | 241 | 0.89 | 298 | 0.11 | 1.292.79 |  |
| Chitosan-GO | CTS/GO-10 | 374 | 1.31 | 298 | 0.11 | 1.443.48 |  |
| Chitosan-GO | CTS/GO-15 | 390 | 1.14 | 298 | 0.11 | 1.733.96 |  |
| Chitosan-GO | CTS/GO-20 | 412 | 1.23 | 298 | 0.11 | 2.114.15 |  |
| Chitosan-GO | CTS/GO-1 | 182 | 0.76 | 322.99 | 1 | 1.71 |  |
| Chitosan-GO | CTS/GO-1 | 182 | 0.76 | 347.86 | 1 | 1.32 |  |
| Chitosan-GO | CTS/GO-3 | 205 | 0.78 | 322.99 | 1 | 1.81 |  |
| Chitosan-GO | CTS/GO-3 | 205 | 0.78 | 347.86 | 1 | 1.4 |  |
| Chitosan-GO | CTS/GO-5 | 241 | 0.89 | 322.99 | 1 | 2.17 |  |
| Chitosan-GO | CTS/GO-5 | 241 | 0.89 | 347.86 | 1 | 1.88 |  |
| Chitosan-GO | CTS/GO-10 | 374 | 1.31 | 323 | 0.11 | 0.92.56 |  |
| Chitosan-GO | CTS/GO-10 | 374 | 1.31 | 348 | 0.11 | 1.442.13 |  |
| Chitosan-GO | CTS/GO-15 | 390 | 1.14 | 323.06 | 1 | 3.19 |  |
| Chitosan-GO | CTS/GO-15 | 390 | 1.14 | 348.08 | 1 | 2.85 |  |
| Chitosan-GO | CTS/GO-20 | 412 | 1.23 | 323.06 | 1 | 3.39 |  |
| Chitosan-GO | CTS/GO-20 | 412 | 1.23 | 343.08 | 1 | 3.07 |  |
| Chitosan-GO | CTS/GO | 33.32 | 0.13 | 298 | 0.94 | 0.25 |  |
| Chitosan-GO |  | 33.32 | 0.13 | 298 | 0.89 | 0.19 |  |
| Chitosan-GO |  | 33.32 | 0.13 | 298 | 0.79 | 0.16 |  |
| Chitosan-GO |  | 33.32 | 0.13 | 298 | 0.69 | 0.12 |  |
| Chitosan-GO |  | 33.32 | 0.13 | 298 | 0.6 | 0.09 | 19 |
| Chitosan-GO |  | 33.32 | 0.13 | 298 | 0.5 | 0.07 |  |
| Chitosan-GO |  | 33.32 | 0.13 | 298 | 0.4 | 0.05 |  |
| Chitosan-GO |  | 33.32 | 0.13 | 298 | 0.3 | 0.03 |  |
| Chitosan-GO |  | 33.32 | 0.13 | 298 | 0.2 | 0.01 |  |
| Composite Porous Monolithic rGO/Polymer | M45-1-10 | 110 | 0.2 | 298 | 1 | 3.85 |  |
| Composite Porous Monolithic rGO/Polymer | M45-1-40 | 67 | 0.15 | 298 | 1 | 1.07 |  |
| Composite Porous Monolithic rGO/Polymer | M45-2-10 | 235 | 0.45 | 298 | 1 | 3.08 |  |
| Composite Porous Monolithic rGO/Polymer | M45-2-40 | 122 | 0.51 | 298 | 1 | 1.76 |  |
| Composite Porous  Monolithic rGO/Polymer | M60-1-10 | 217 | 0.63 | 298 | 1 | 3.56 |  |
| Composite Porous  Monolithic rGO/Polymer | M60-1-40 | 154 | 0.6 | 298 | 1 | 1.37 |  |
| Composite Porous  Monolithic rGO/Polymer | M60-2-10 | 211 | 0.55 | 298 | 1 | 1.65 | 56 |
| Composite Porous  Monolithic rGO/Polymer | M60-2-40 | 165 | 0.52 | 298 | 1 | 1.26 |  |
| Composite Porous  Monolithic rGO/Polymer | M90-1-10 | 171 | 0.47 | 298 | 1 | 3.79 |  |
| Composite Porous  Monolithic rGO/Polymer | M90-1-40 | 203 | 0.71 | 298 | 1 | 1.16 |  |
| Composite Porous  Monolithic rGO/Polymer | M90-2-10 | 244 | 0.83 | 298 | 1 | 1.84 |  |
| Composite Porous  Monolithic rGO/Polymer | M90-2-40 | 188 | 0.71 | 298 | 1 | 1.25 |  |
| copper-based metal-organic framework and graphite oxide composite | HKUST-1/GO | 1015.25 | 0.5 | 305 | 15 | 0.362.48 | 57 |
| MOF(CU)-graphene oxide | CuBTC/GO-0.5 | 1675 | 0.69 | 273 | 0.051 | 0.78.33 | 54 |
| MOF(CU)-graphene oxide | CuBTC/GO-1 | 1772 | 0.72 | 273 | 0.051 | 0.648.98 |  |
| MOF(CU)-graphene oxide | CuBTC/GO-3 | 1668 | 0.7 | 273 | 0.051 | 0.78.33 |  |
| MOF(CU)-graphene oxide | CuBTC/GO-5 | 1637 | 0.67 | 273 | 0.051 | 0.668.34 |  |
| UiO-66/graphene oxide composites | UiO-66/GO-1 | 923 | 0.31 | 298 | 0.151.11 | 0.512.66 |  |
| UiO-66/graphene oxide composites | UiO-66/GO-5 | 1184 | 0.38 | 298 | 0.11.11 | 0.733.74 | 58 |
| UiO-66/graphene oxide composites | UiO-66/GO-10 | 1012 | 0.41 | 298 | 0.071.11 | 0.433.33 |  |
| Monolith-Temperature-  GO:AsA(Ascorbic Acid) | M45_1 | 170 | 0.39 | 298 | 0.121 | 0.151.2 |  |
| Monolith-Temperature-  GO:AsA(Ascorbic Acid) | M60_1 | 166 | 0.33 | 298 | 0.181.01 | 0.110.57 |  |
| Monolith-Temperature-  GO:AsA(Ascorbic Acid) | M90_1 | 319 | 1.16 | 298 | 0.131.02 | 0.141.41 |  |
| Monolith-Temperature-  GO:AsA(Ascorbic Acid) | M60_0.5 | 60 | 0.14 | 298 | 0.121 | 0.221.14 | 59 |
| Monolith-Temperature-  GO:AsA(Ascorbic Acid) | M90_0.5 | 328 | 1.35 | 298 | 0.131 | 0.181.91 |  |
| Monolith-Temperature-  GO:AsA(Ascorbic Acid) | M60_2 | 220 | 0.71 | 298 | 0.131.01 | 0.090.71 |  |
| Monolith-Temperature-  GO:AsA(Ascorbic Acid) | M90_2 | 166 | 0.66 | 298 | 0.131.01 | 0.090.84 |  |
| mesoporous TiO2/graphene oxide nanocomposites | TiO2/GO-0.1 | 99.54 | 0.38 | 273 | 0.080.96 | 0.42.19 |  |
| mesoporous TiO2/graphene oxide nanocomposites | TiO2/GO-0.2 | 87.77 | 0.3 | 273 | 0.150.96 | 0.421.56 |  |
| mesoporous TiO2/graphene oxide nanocomposites | TiO2/GO-0.3 | 83.12 | 0.27 | 273 | 0.180.96 | 0.41.14 | 51 |
| mesoporous TiO2/graphene oxide nanocomposites | TiO2/GO-0.1 | 99.54 | 0.38 | 298 | 0.081 | 0.231.82 |  |
| polypyrrole-derived carbon/GO composites | aPPy/GO 5% | 2560 | 1.22 | 273298 | 1 | 7.284.14 |  |
| polypyrrole-derived carbon/GO composites | aPPy/GO 10% | 2640 | 1.28 | 273298 | 1 | 7.144.16 |  |
| polypyrrole-derived carbon/GO composites | aPPy/GO 20% | 2270 | 1.1 | 273298 | 1 | 6.753.86 | 22 |
| polyaniline-derived carbon/GO composites | aPAni/GO-5 | 1910 | 0.87 | 273298 | 1 | 7.114.27 |  |
| polyaniline-derived carbon/GO composites | aPAni/GO-10 | 1770 | 0.91 | 273298 | 1 | 6.834.11 |  |
| Cu-containing metal-organic framework/GO composites | CuBTC/GO-2 | 1820 | 0.88 | 273298 | 1 | 9.055.12 |  |
| Cu-containing metal-organic framework/GO composites | CuBTC/GO-5 | 1520 | 0.78 | 273298 | 1 | 8.464.79 | 22 |
| Cu-containing metal-organic framework/GO composites | CuBTC/GO-10 | 1380 | 0.73 | 273298 | 1 | 7.024.11 |  |
| Cu-containing metal-organic framework/GO composites | CG-3 | 1470 | 0.61 | 273 | 0.011 | 0.087.94 |  |
|  | CG-9 | 1532 | 0.64 | 273 | 0.011 | 0.078.26 | 52 |
|  | CG-15 | 500 | 0.31 | 273 | 0.011 | 0.092.97 |  |
| graphene oxide/ordered mesoporous carbon composite Ordered | GO5/aOMC | 1370 | 1.06 | 273 | 0.071 | 1.255.64 | 60 |
|  | GO/core shell ZIF-8@ZIF-67-0.5 | 1378 | 1.27 | 273 | 0.231 | 0.412.15 |  |
|  | GO/core shell ZIF-8@ZIF-67-1 | 1056 | 0.83 | 273 | 0.311 | 0.451.72 | 61 |
|  | GO/core shell ZIF-8@ZIF-67-2 | 1003 | 0.7 | 273 | 0.271 | 0.381.55 |  |
|  | GO/core shell ZIF-8@ZIF-67-4 | 585 | 0.82 | 273 | 0.21 | 0.25-1.3 |  |
| PEI/GO: 3:1 | GEMP -1 | 253 | 0.7 | 273 | 0.021.05 | 0.732.48 |  |
| PEI/GO: 2:1 | GEMP -2 | \| 354 \| 273 \| 1.1 \| 1 \| 1.9 \| \| --- \| --- \| --- \| --- \| --- \| | 1.1 | 273 | 1 | 1.9 | 50 |
| PEI/GO: 1:1 | GENP-3 | 476 | 1.3 | 273 | 1 | 2.2 |  |
| PEI/GO: 1:3 | GEMP-4 | 455 | 1.2 | 273 | 1 | 1.65 |  |
| Graphene-manganese oxide hybrid porous | GMNO-1 | 140 | 0.12 | 273 | 1 | 0.45 | 62 |
|  | GMNO-2 | 283 | 0.19 | 273 | 1 | 0.77 |  |
|  | GMNO-3 | 410 | 0.26 | 273 | 1 | 1.98 |  |
|  | GMNO-4 | 541 | 0.31 | 273 | 0.131 | 0.442.59 |  |
|  | GMNO-5 | 685 | 0.48 | 273 | 1 | 1.3 |  |
| Chitosan grafted graphene Oxide | CS-GO | 33.32 | 0.13 | 273 | 0.21 | 0.010.25 | 19 |
| amine- modified graphite oxides (GAs) prepared | GA-W | 9.6 | 0.03 | 273 | 0.051 | 0.782.26 |  |
|  | GA-W | 9.6 | 0.03 | 303 | 0.071 | 0.411.49 |  |
|  | GA-W | 9.6 | 0.03 | 323 | 0.091 | 0.320.97 | 63 |
|  | GA-E | 10.2 | 0.03 | 273 | 0.051 | 0.882 |  |
|  | GA-E | 10.2 | 0.03 | 303 | 0.071 | 0.321.28 |  |
|  | GA-E | 10.2 | 0.03 | 323 | 0.091 | 0.170.79 |  |
| amorphous carbon and MgO nano crystallites grafted on reduced graphene oxide | rGO@MgO/C-2 | 357.7 | 0.88 | 300 | 0.151 | 56.42 |  |
| amorphous carbon and MgO nano crystallites grafted on reduced graphene oxide | rGO@MgO/C-5 | 478.4 | 1.22 | 300 | 0.151 | 5.117.05 | 64 |
| amorphous carbon and MgO nano crystallites grafted on reduced graphene oxide | rGO@MgO/C-10 | 463.9 | 1.25 | 300 | 0.151 | 4.96.22 |  |
| amorphous carbon and MgO nano crystallites grafted on reduced graphene oxide | rGO@MgO/C-15 | 318.2 | 0.58 | 300 | 0.151 | 4.544.97 |  |
|  | IFGO | 190 | .096 | 273,298 | 0.52.7 | 0.497.94 | 65 |

**References**

19 Hsan, N., Dutta, P. K., Kumar, S., Bera, R. & Das, N. Chitosan grafted graphene oxide aerogel: Synthesis, characterization and carbon dioxide capture study. *International Journal of Biological Macromolecules* **125**, 300-306, (2019).

22 Szczęśniak, B. & Choma, J. Graphene-containing microporous composites for selective CO2 adsorption. *Microporous and Mesoporous Materials* **292**, 109761, (2020).

50 Sui, Z.-Y., Cui, Y., Zhu, J.-H. & Han, B.-H. Preparation of Three-Dimensional Graphene Oxide–Polyethylenimine Porous Materials as Dye and Gas Adsorbents. *ACS Applied Materials & Interfaces* **5**, 9172-9179, (2013).

51 Chowdhury, S., Parshetti, G. K. & Balasubramanian, R. Post-combustion CO2 capture using mesoporous TiO2/graphene oxide nanocomposites. *Chemical Engineering Journal* **263**, 374-384, (2015).

52 Liu, S. *et al.* Nanosized Cu-MOFs induced by graphene oxide and enhanced gas storage capacity. *Energy & Environmental Science* **6**, 818-823, (2013).

53 Alhwaige, A. A., Agag, T., Ishida, H. & Qutubuddin, S. Biobased chitosan hybrid aerogels with superior adsorption: Role of graphene oxide in CO2 capture. *RSC Advances* **3**, 16011-16020, (2013).

54 Shang, S. *et al.* Facile synthesis of CuBTC and its graphene oxide composites as efficient adsorbents for CO2 capture. *Chemical Engineering Journal* **393**, 124666, (2020).

56 Politakos, N. *et al.* Reduced Graphene Oxide/Polymer Monolithic Materials for Selective CO2 Capture. *Polymers* **12**, (2020).

57 Zhao, Y., Cao, Y. & Zhong, Q. CO2 capture on metal-organic framework and graphene oxide composite using a high-pressure static adsorption apparatus. *J. Clean Energy Technol* **2**, 34-37, (2014).

58 Cao, Y., Zhao, Y., Lv, Z., Song, F. & Zhong, Q. Preparation and enhanced CO2 adsorption capacity of UiO-66/graphene oxide composites. *Journal of Industrial and Engineering Chemistry* **27**, 102-107, (2015).

59 Politakos, N. *et al.* Graphene-Based Monolithic Nanostructures for CO2 Capture. *Industrial & Engineering Chemistry Research* **59**, 8612-8621, (2020).

60 Szczęśniak, B., Choma, J. & Jaroniec, M. Effect of graphene oxide on the adsorption properties of ordered mesoporous carbons toward H2, C6H6, CH4 and CO2. *Microporous and Mesoporous Materials* **261**, 105-110, (2018).

61 Liu, N., Cheng, J., Hou, W., Yang, X. & Zhou, J. Unsaturated Zn–N2–O active sites derived from hydroxyl in graphene oxide and zinc atoms in core shell ZIF-8@ZIF-67 nanocomposites enhanced CO2 adsorption capacity. *Microporous and Mesoporous Materials* **312**, 110786, (2021).

62 Zhou, D. *et al.* Graphene-manganese oxide hybrid porous material and its application in carbon dioxide adsorption. *Chinese Science Bulletin* **57**, 3059-3064, (2012).

63 Hong, S. M. & Lee, K. B. Solvent-assisted amine modification of graphite oxide for CO2 adsorption. *RSC Advances* **4**, 56707-56712, (2014).

64 Li, P. & Zeng, H. C. Hierarchical Nanocomposite by the Integration of Reduced Graphene Oxide and Amorphous Carbon with Ultrafine MgO Nanocrystallites for Enhanced CO2 Capture. *Environmental Science & Technology* **51**, 12998-13007, (2017).

65 Bhanja, P., Das, S. K., Patra, A. K. & Bhaumik, A. Functionalized graphene oxide as an efficient adsorbent for CO2 capture and support for heterogeneous catalysis. *RSC Advances* **6**, 72055-72068, (2016).
